# Virus Discovery in Desert Tortoise Fecal Samples: Novel Circular Single-Stranded DNA Viruses

**DOI:** 10.3390/v12020143

**Published:** 2020-01-26

**Authors:** Joseph P. Orton, Matheo Morales, Rafaela S. Fontenele, Kara Schmidlin, Simona Kraberger, Daniel J. Leavitt, Timothy H. Webster, Melissa A. Wilson, Kenro Kusumi, Greer A. Dolby, Arvind Varsani

**Affiliations:** 1 School of Life Sciences, Arizona State University, Tempe, AZ 85287, USA; jporton@asu.edu (J.P.O.); mmoral31@asu.edu (M.M.); rafasfontenele@asu.edu (R.S.F.); Kara.Schmidlin@asu.edu (K.S.); mwilsons@asu.edu (M.A.W.); Kenro.Kusumi@asu.edu (K.K.); 2 The Biodesign Center for Fundamental and Applied Microbiomics, Arizona State University, Tempe, AZ 85287, USA; simona.kraberger@asu.edu; 3 Natural Resources Program, Naval Facilities Engineering Command-Navy Region Southwest, San Diego, CA 92101, USA, USA; daniel.j.leavitt@navy.mil; 4 Department of Anthropology, University of Utah, Salt Lake City, UT 84112, USA; 5 Center for Evolution and Medicine, Arizona State University, Tempe, AZ 85287, USA; 6 Structural Biology Research Unit, Department of Clinical Laboratory Sciences, University of Cape Town, Cape Town 7925, South Africa

**Keywords:** Arizona, *Genomoviridae*, *Microviridae*, CRESS DNA viruses, *Gopherus morafkai*

## Abstract

The Sonoran Desert tortoise *Gopherus morafkai* is adapted to the desert, and plays an important ecological role in this environment. There is limited information on the viral diversity associated with tortoises (family Testudinidae), and to date no DNA virus has been identified associated with these animals. This study aimed to assess the diversity of DNA viruses associated with the Sonoran Desert tortoise by sampling their fecal matter. A viral metagenomics approach was used to identify the DNA viruses in fecal samples from wild Sonoran Desert tortoises in Arizona, USA. In total, 156 novel single-stranded DNA viruses were identified from 40 fecal samples. Those belonged to two known viral families, the *Genomoviridae* (*n* = 27) and *Microviridae* (*n* = 119). In addition, 10 genomes were recovered that belong to the unclassified group of circular-replication associated protein encoding single-stranded (CRESS) DNA virus and five circular molecules encoding viral-like proteins.

## 1. Introduction

The Sonoran Desert tortoises (*Gopherus morafkai*) are long-lived animals (>50 years in the wild) [1,2] adapted to the Mojave and Sonoran deserts of southwestern North America. Spending much of their time underground in burrows or shelters, including months spent brumating during winter [3,4], they interact closely with desert soils and have commensal relationships with many desert animals (e.g., ground squirrels, wood rats, snakes, and spiders) through shared use of shelters [5]. The Sonoran Desert tortoises eat a wide range of native desert grasses [6], are active in the summer monsoon [7], occupy rocky hillsides and streambeds laden with caliche caves [8], and usually use existing rock or caliche shelters [7]. They are thought to have speciated in isolation from Mojave desert tortoise (*Gopherus agassizii*) approximately 5 MYA when the Colorado River bisected the ancestral population and began flowing into the Gulf of California [7,8]. Differences in the timing and amount of rainfall between the Mojave and Sonoran deserts may also have led to differential adaptation between these species over the same time period [9].

The Sonoran Desert tortoise does not appear to face the health-related effects of upper respiratory tract disease observed in Mojave desert tortoise caused by infectious bacterium *Mycoplasma agassizii* [10], perhaps resulting from some inherently different immunological response, lower frequency of encountering the pathogen, or healthier populations due to higher genetic diversity and census size [11,12,13]. There is limited information about other pathogens infecting the tortoise family Testudinidae. So far, viruses from several families have been identified as infecting members of Testudinidae, including picornaviruses [14,15], iridoviruses [16], herpesviruses [17], adenoviruses [18,19,20,21], paramyxoviruses [22,23], and the retrovirus Rous sarcoma virus [24]. There is no information on single-stranded DNA (ssDNA) viruses associated with members of the Testudinidae family and limited viral disease information on the Sonoran Desert tortoise (*G. morafkai*).

We used non-invasive fecal sampling coupled with a viral metagenomic approach to identify the circular ssDNA virus diversity associated with the Sonoran Desert tortoise. We identified novel ssDNA viruses that belong to the *Genomoviridae* [25] and *Microviridae* [26] families. In addition, we identified a suite of unclassified circular replication-associated protein (Rep) encoding single-stranded (CRESS) DNA viruses DNA viruses and small ssDNA molecules encoding viral like proteins. CRESS DNA viruses include viruses in the families *Bacilladnaviridae* [27], *Circoviridae* [28], *Geminiviridae* [29], *Genomoviridae* [25], and *Nanoviridae* [30] and *Smacoviridae* [31,32]. Through viral metagenomics approaches, a larger number of novel CRESS DNA viruses have been identified that do not fall within the established viral taxonomy framework and thus are loosely referred to as ‘unclassified CRESS DNA viruses’.

## 2. Materials and Methods

### 2.1. Sample Collection and Processing

Sonoran Desert tortoise fecal samples (*n* = 40) were collected in the Sonoran Desert of Arizona, USA, in 2013 and 2014. Samples were collected in the field from five locations in Maricopa, Yavapai, Pinal, and La Paz Counties, stored in separate plastic bags, and frozen once returned to the field station. DNA was extracted as previously described by Steel et al. [33]. Briefly, approximately 5 g of fecal material per sample was homogenized in 20 mL SM buffer (0.1 M NaCl, 50 mM Tris-HCl [pH 7.4], 10 mM MgSO_4_) and centrifuged for 4700× *g* for 15 min. The supernatant was filtered sequentially through a 0.45 µm, and 0.22 µm syringe filter. Three grams of PEG 8000 (Sigma, St. Louis, MO, USA) was added to 15 mL of each of the filtrates, the solution was mixed gently to suspend the PEG and incubated overnight at 4 °C to precipitate virions. The mixture was centrifuged at 15,000× *g* for 20 min and the resulting pellet was resuspended in 1 mL of filtrate. Viral DNA was extracted from 200 µL of resuspension using the Roche High Pure Viral nucleic acid kit (Roche Diagnostics, Indianapolis, IN, USA). 

### 2.2. Illumina Sequencing and Data Processing

Circular molecules were amplified using rolling circle amplification (RCA) with the Illustra TempliPhi 100 Amplification Kit (GE Healthcare, Chicago, IL, USA). The RCA products of the 40 samples were pooled into five groups each with 6–9 samples. These pools were used to generate a 2 × 100 bp library and sequenced on a HiSeq 4000 platform at Macrogen, Inc. (Seoul, Korea). The raw paired-end reads were trimmed using Trimmomatic v0.36 [34] and *de novo* assembled using metaSPAdes 3.12.0 [35]. Circular contigs were identified based on terminal sequence redundancy and all contigs >500 nucleotides (nt) were analyzed using BLASTx [36] against a local viral RefSeq protein database (complete database downloaded on 15 July 2019) compiled from GenBank. 

### 2.3. Recovery of Complete Genomes of Genomoviruses and Unclassified CRESS DNA Viruses

Abutting primers were designed (Supplementary Table S1) based on the de novo assembled contigs for genomoviruses and unclassified CRESS DNA. These were used for PCR amplification of full genomes. For each PCR amplification, 1 µL of RCA product from each pooled sample was used as a template with primer pairs and Kapa HiFi Hotstart Ready Mix (Kapa Biosystems, Wilmington, MA, USA) using the following thermal cycling conditions: initial denaturation at 95 °C for 3 min, followed by 25 cycles at 98 °C for 20 s, 60 °C for 15 s and 72 °C for 3 min, final elongation at 72 °C for 3 min and a final renaturation at 4 °C for 10 min. The amplicons were resolved on a 0.7% agarose gel, amplicons of ~1.5–3 kb were excised, and gel purified, ligated in pJET1.2 vector (ThermoFisher Scientific, Waltham, MA, USA) and transformed into *Escherichia coli* XL1-Blue competent cells. The recombinant plasmids were Sanger sequenced at Macrogen Inc. (Seoul, Korea) by primer walking and these sequence contigs were assembled using Geneious 11.1 [37]. 

### 2.4. Viral Sequence Analysis

All the complete microvirus genomes from the *de novo* assemblies were checked by mapping processed raw reads to the assembled genomes using BBMap v 38.32 [38]. All the open reading frames for genomoviruses and the unclassified CRESS DNA viruses were determined using ORF finder (https://www.ncbi.nlm.nih.gov/orffinder/) coupled with manual determination of splice sites for the Reps. For the microviruses, the ORFs were identified using Glimmer [39]. All genome and encoded protein sequence pairwise identities were determined using SDT v1.2 [40]. 

A dataset of Reps assembled in Fontenele et al. [41] was used together with the Rep protein sequences identified in this study to construct a sequence similarity network (SSN) using EFI-EST [42,43] with a similarity score of 60 that allow for clear viral family-level clustering. The SSN was visualized using the organic layout in Cytoscape V3.7.1 [44].

### 2.5. Phylogenetic Analysis

#### 2.5.1. Genomoviruses

The Rep protein sequences of the genomoviruses were aligned using MUSCLE [45] and the resulting alignment was used to infer a Maximum-Likelihood phylogenetic tree using PhyML 3.0 [46] with rtREV+G+I+F substitution model (inferred as best fit model using ProtTest [47]) with approximate likelihood ratio test (aLRT) for branch support. The genomovirus Rep amino acid sequence maximum likelihood phylogenetic tree which was rooted with geminivirus Rep sequences. The phylogenetic tree was visualized using iTOL [48].

#### 2.5.2. Unclassified CRESS DNA Viruses

For the unclassified CRESS DNA viruses, Reps encoded by viruses identified in this study falling within clusters from the SSN analysis of ≥5 sequences were aligned with sequences that were part of that cluster using MUSCLE [45]. and Maximum-Likelihood phylogenetic trees were inferred from these alignments using PhyML 3.0 [46] with rtREV+G substitution model for the three phylogenetic trees based on results from ProtTest [47] with approximate likelihood ratio test (aLRT) for branch support. The phylogenetic trees were midpoint rooted and branches with <0.8 aLRT support were collapsed using TreeGraph2 [49]. All the phylogenetic trees were visualized using iTOL [48].

#### 2.5.3. Microviruses

The MCPs of 2590 microviruses available in GenBank, 88 from metagenomics studies described in Roux et al. [50] and Krupovic and Forterre [51], and 119 (Supplementary Data 1) from this study were aligned using PROMAL3D [52,53]. The resulting alignment was used to infer an approximately-Maximum-Likelihood phylogenetic tree using FastTree 2 [54]. The resulting tree was rooted with MCPs of viruses in the family *Bullavirinae*, and visualized and annotated using iTOL [48].

### 2.6. Recombination Analysis of Genomoviruses

The full genome alignments, aligned using MAFFT [55], of gemycircularviruses (*n* = 208), gemykibiviruses (*n* = 123) and gemykoloviruses (*n* = 51) were used to identify evidence of recombination using RDP 4 [56]. The recombination analysis was run with default settings using the detection methods RDP [57], GENECONV [58], BOOTSCAN [59], MAXCHI [60], CHIMERA [61], SISCAN [62] and 3SEQ [63]. Recombination events that were detected by three or more methods with *p*-values < 0.05 coupled with phylogenetic support were considered credible.

## 3. Results and Discussion

### 3.1. Identification of ssDNA Virus Genomes in Sonoran Desert Tortoise Fecal Samples

Using a viral metagenomic approach, we identified 156 unique complete genomes of ssDNA viruses from 40 fecal samples of Sonoran Desert tortoises. Of these, 27 are unique genomoviruses, 119 are unique microviruses and 10 are unique unclassified CRESS DNA viruses. In addition, we identified four unique Rep-encoding and one unique CP-encoding circular molecules. 

A hallmark of all Rep proteins of CRESS DNA viruses are the conserved rolling-circle replication (RCR) endonuclease and superfamily 3 (SF3) helicase domains [64,65]. In 39 of the 41 Rep-encoding viruses/molecules from this study, we were able to identify the three RCR motifs (Motif I, II and II) and the three SF3 helicase motifs (Walker A, Walker B, and Motif C) (Table 1). For the unclassified CRESS DNA viruses M858258, we were unable to identify the entire RCR domain and for the Rep-encoding circular molecule MK858264 we were unable to identify Motif I of the RCR domain. 

### 3.2. Genomoviruses

*Genomoviridae* is a recently established family of diverse circular ssDNA viruses [66]. Genomoviruses have an ambisense genome organization and genomes that are ~ 1.9–2.3 kb encoding a CP on the virion sense and Rep on the complementary sense. The family *Genomoviridae* is divided into nine genera (*Gemycircularvirus*, *Gemyduguivirus*, *Gemygorvirus*, *Gemykibivirus*, *Gemykolovirus*, *Gemykrogvirus*, *Gemykroznavirus*, *Gemytondvirus* and *Gemyvongvirus*) [25]. In general, the genomoviruses are classified at a species level based on their genome-wide pairwise identity with a species cutoff threshold of 78%. Even through genomoviruses have been identified from various sources (animal fluid, tissue and fecal samples, wastewater, river sediments and plant material), Sclerotinia sclerotiorum hypovirulence-associated DNA virus 1 (SsHADV-1) is the only genomovirus with a known host, the fungi *Sclerotinia sclerotiorum* in which it induces hypovirulence [67]. Thus, it is highly likely that genomoviruses viruses infect fungi. 

**Figure 1:**
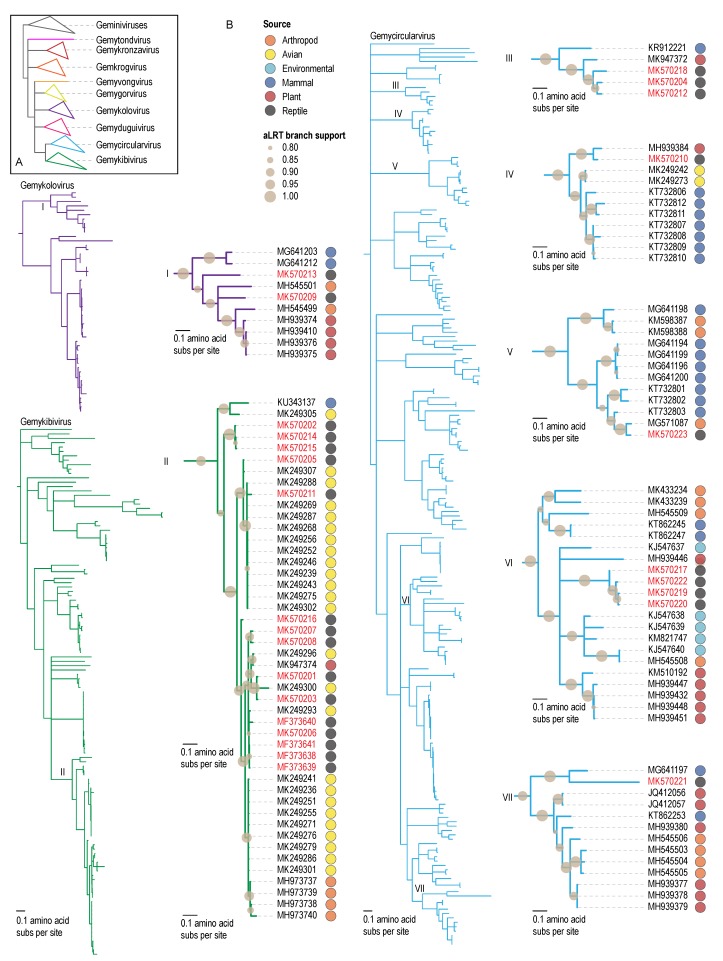
**A.** Phylogenetic relationship of the Rep protein sequences of genomoviruses with genus level clustering. **B**. Maximum likelihood phylogenetic trees of the Reps sequences of genomoviruses identified in this study that belong to the genera *Gemykolovirus*, *Gemykibivirus* and *Gemycircularvirus*. For each tree, a detailed view of the clusters that have the Reps from this study is provided to the right of the genus level trees. Next to each accession number the source of the sequence is provided.

The tortoise-associated genomoviruses (*n* = 27) identified in this study can be assigned to three of the nine genera, i.e., *Gemycircularvirus* (*n* = 10), *Gemykibivirus* (*n* = 15) and *Gemykolovirus* (*n* = 2) based on their Rep amino acid sequence (Figure 1). The 10 gemycircularviruses identified in tortoise feces share 66%–98% genome-wide nucleotide pairwise identity between themselves and 65%–83% with other gemycircularvirus sequences in GenBank. These tortoise genomoviruses can be further classified as belonging to six new species, all of which would need to be established based on the criteria outlined for the classification of genomoviruses (i.e., 78% pairwise identity threshold) [25]. Even though MK570223 shares ~82.5% nucleotide identity with a gemycircularvirus identified from Varroa mite samples from New Zealand [68], this virus has not been yet classified and together they would represent a new species. The 15 gemykibivirus identified in tortoise feces share >94% nucleotide pairwise identity amongst themselves and >90% with gemykibiviruses identified from house finch feces and nests from Arizona [69], as a collective these represent new species. The two gemykoloviruses share ~65% nucleotide pairwise identity with each other and 66%–70% with other gemykolovirus sequences in GenBank, and they represent two new species. A summary of the gemycircularvirus (*n* = 10), gemykibivirus (*n* = 15) and gemykolovirus (*n* = 2) virus Rep and CP amino acid sequence pairwise identities between themselves and those encoded by genomovirus sequences in GenBank are provided in Table 2.

**Figure 2:**
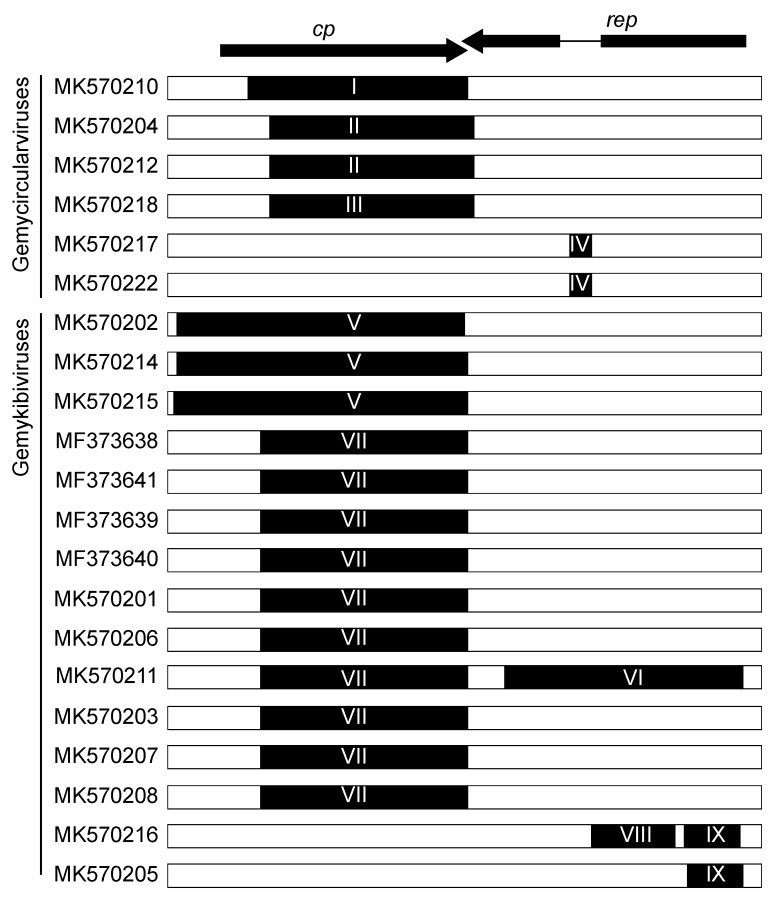
Illustration of the nine events of recombination detected by RDP4 in the genomes of gemycircularviruses (*n* = 6) and gemykibiviruses (*n* = 15). The black regions depict the recombinant region, with I–IX denoting the recombination events (see Table 3 for details of recombination). The genome organization of genomoviruses is provided at the top showing the *cp* and *rep* genes.

We identified nine events of recombination in the genomoviruses from this study, four events in six genomes of gemycircularviruses and five events in 15 genomes of gemykibiviruses (Figure 2 and Table 3). Five of the nine recombinant regions span most of the *cp* gene (766–1121 nt) and one (917 nt) the *rep* gene. Three small recombinant regions were identified in the *rep* genes spanning 52–315 nt (Figure 2 and Table 3). In the case of gemykibivirus genome (MK570211), ~76% of the genome is a recombinant. We found no evidence of recombination in the genomes of the two gemykoloviruses.

### 3.3. Unclassified Eukaryotic CRESS DNA Viruses and Circular DNA Molecules

**Figure 3:**
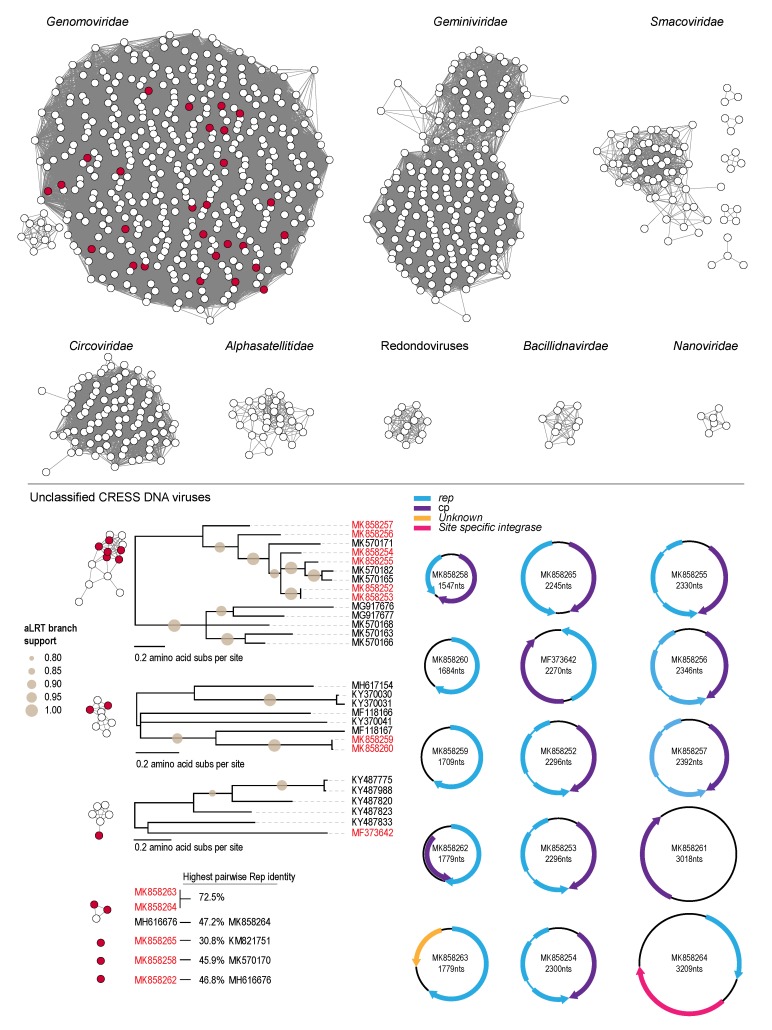
Sequence similarity networks showing family-level clustering of the Reps of CRESS DNA viruses and Rep-encoding circular molecules. For each cluster that has a Rep from this study (colored in red), a phylogenetic tree (midpoint rooted) has been inferred for all sequences belonging to the cluster.

Over the last decade, there has been a significant number of novel CRESS DNA viruses that have been discovered in various environments. This has primarily been facilitated by viral metagenomic studies using high throughput sequencing approaches. Most of these novel CRESS DNA viruses cannot be classified into established viral families and thus are referred to as unclassified CRESS DNA viruses. Here, we identified 10 CRESS DNA viruses ranging in size from 1547 to 2300 nt (Figure 3). Given the large number of unclassified CRESS DNA viruses (>2000), we used an SSN based approach to cluster the Rep sequences of those that are classified with those from this study (Figure 3). With a SSN threshold of 60, we are able to generate family-level clusters which support currently classified viruses, i.e., *Bacilladnaviridae*, *Circoviridae*, *Geminiviridae*, *Genomoviridae*, *Nanoviridae* and *Smacoviridae*, as well as *Alphasatellitidae* and a recently proposed family-level group of viruses called redondoviruses (Figure 3). The only Rep amino acid sequences of viruses identified in this study that cluster with any of these are these are those of genomoviruses. All other Reps of CRESS DNA viruses and Rep-encoding circular molecules cluster form four clusters and three are singletons. 

Six of the unclassified CRESS DNA viruses (MK858252-MK858257) have a similar genome organization, i.e., their CP is encoded on the virion sense and their Rep (that has two predicted introns) on the complementary sense. These six genomes share >62% genome-wide nucleotide identity and all have a ‘TAAGATTAC’ nonanucleotide motif. Their Reps share >56% amino acid identity, whereas their CPs share >54% amino acid identity. These Rep amino acid sequences cluster in the SSN and phylogenetically with Reps of viruses from a termite mound (sampled in Kenya) [70] and capybara feces (sampled in Brazil) [41] (Figure 3). The Reps amino acid sequences of the circular molecules MK858259 and MK858260 are most closely related to that of MF118167 from a human fecal sample [71] sharing ~49% amino acid identity whereas the Rep of MF373642 shares 42% amino acid identify with that of KY487833 from wastewater [72] and cluster with other wastewater derived sequences (Figure 3; Table 4). Sequences of MF373642, MK858258, MK858262 and MK858265 encode Reps that share 30%–46% amino acid identity with other unclassified CRESS DNA virus Reps. 

In addition to the unclassified CRESS DNA viruses, five circular molecules ranging in size from 1684 to 3209 nt were also identified. Four of these five encode at least a Rep and one encodes only a CP (which shares ~52% amino acid identity with that of MK858258). Of the four that encode a Rep, one circular molecule (MK858264) also encodes a site-specific integrase sharing 62% identity (99% coverage; E-Value 9×10^-175^) with one from an *Oscillibacter* sp. (CDB27191).

### 3.4. Microviruses

Microviruses are bacteriophages and the family *Microviridae* is divided into two subfamilies, *Bullavirinae* and *Gokushovirinae* [26]. Microviruses that have been well studied are known to infect enterobacteria. Thus, the detection of these viruses in the tortoise fecal samples is highly likely to be associated with their gut microbial flora. Over the last couple of years, there have been a large number of microviruses that have been identified in various sample types from vertebrates, invertebrates, and environmental samples. Despite there being >2500 genomes in GenBank which are very diverse, microviruses are poorly classified at a taxonomic level.

**Figure 4:**
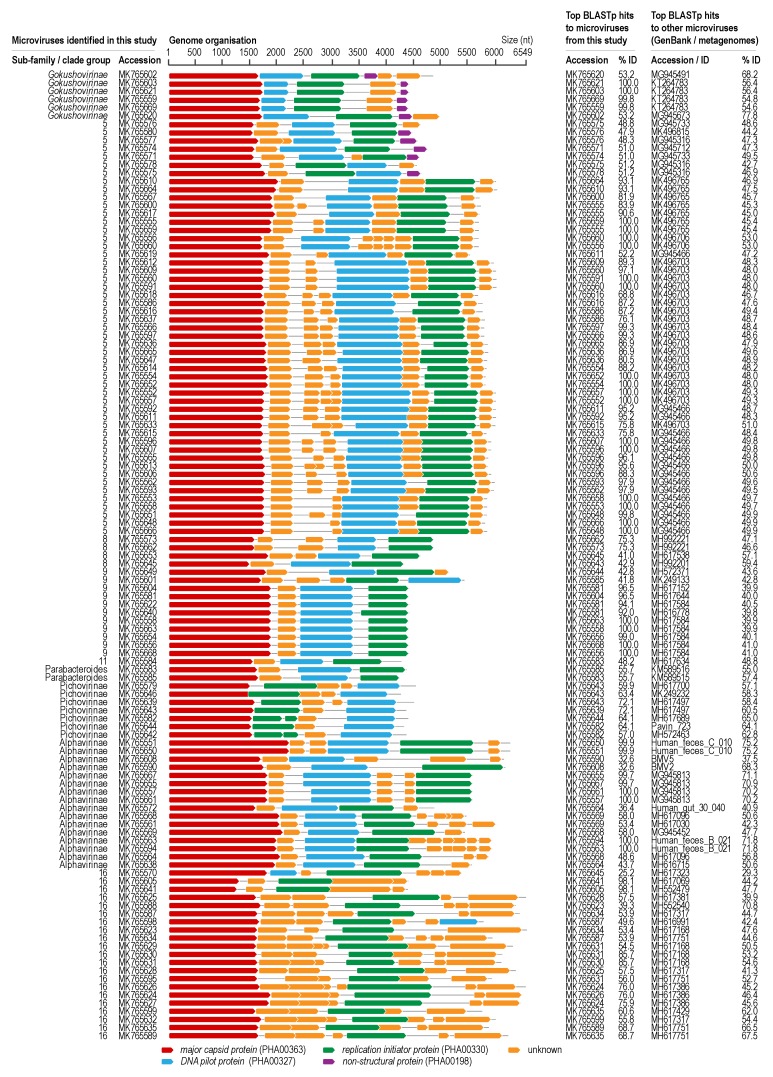
A. Linearized genome representation of the genomes of microviruses identified in this study with color coded open reading frames. B. Pairwise identities of the MCP amino acid sequences (showing highest identity) of the microviruses identified in this study against themselves and against those available in GenBank. *Gokushovirinae* is an officially recognized sub-family of the family *Microviridae*. Alphavirinae, Pichovirinae and Parabacteroides group are proposed groups within the family *Microviridae* [50,51]. Clade group is based on the phylogenetic analysis of the MCPs (Figure 5; Supplementary Data 1).

In this study, we identified 119 genomes (size range 4217–6549 nt) of microviruses from the 40 samples, of which 111 share less than 98% genome wide nucleotide identity (Figure 4). All of these 119 microviruses encode at least a MCP, DNA pilot protein and a replication initiator protein with the exception of MK765635, which appears to be missing a DNA pilot protein. The genomic organization in terms of gene sequence order varies across all the microviruses identified in this study (Figure 4). The ORF coding for the replication initiator protein of MK765582 and MK765642 appears to have an intron. Although introns are rare in bacteria, introns have been identified in bacteriophage ORFs [73].

Of all the proteins encoded by microviruses, the MCP is the most conserved and thus is generally used to determine relationships between these viruses [74]. Of the >2500 genomes of microvirus available in public databases, only a handful have been classified into three genera, *Bdellomicrovirus* (two species), *Chlamydiamicrovirus* (four species) and *Spiromicrovirus* (one species) for *Gokushovirinae*. Similarly, there are only three genera for *Bullavirinae* (*Alphatrevirus*, *Gequatrovirus* and *Sinsheimervirus*). The MCPs of the 119 microviruses identified in this study share 25%–100% amino acid identity amongst themselves and 25%–76.7% with those of previously identified microviruses (Figure 4). 

**Figure 5:**
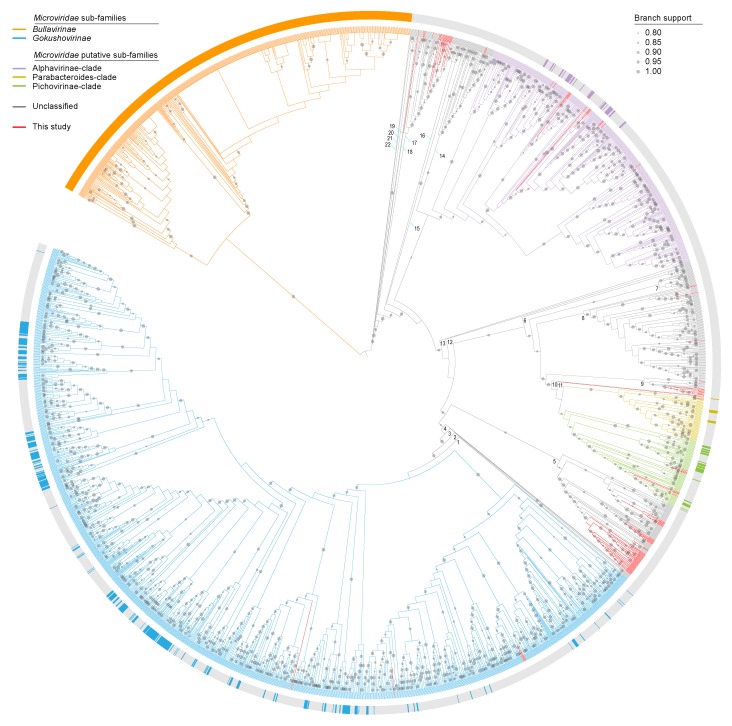
Approximately Maximum-Likelihood cladogram of the MCP sequences (*n* = 2797). Branches are color coded based on sub-families (*Bullavirinae* and *Gokushovirinae*) and Alphavirinae-, Parabacteroides- and Pichovirinae-clades. In addition to these, 22 unique clades are marked with numbers. Branches in grey represent an additional nine singletons and 13 clades. Branches in red denote sequences identified in this study. The outer circle represents taxa with some level of classification assigned prior to this study. Branch support with >0.8 aLRT is shown. Detailed cladogram and phylogram are provided in Supplementary Figures S1 and S2 and taxa names and assignment are provided in Supplementary Data 1.

Beyond the official recognition of two sub-families for *Microviridae*, a handful of clades have be identified that may potentially be considered as sub-families [50,51]. Here, we refer to these as Alphavirinae-, Parabacteroides- and Pichovirinae-clades (Figure 5). Further, there are nine singletons and 13 clades that we have identified that are also unique (Supplementary Data 1). 

Of the 119 microviruses identified here, six belong to the sub-family *Gokushovirinae*, two to Parabacteroides clade, seven to proposed Pichovirinae clade and 14 to Alphavirinae clade. Vast majority of the microviruses from this study fall within clade 5 (*n* = 51) and the remaining in clades 8 (*n* = 4), 9 (*n* = 11), 11 (*n* = 1), 14 (*n* = 2) 17 (*n* = 21) (Supplementary Data 1). The tortoise feces derived microviruses represent 52.4% (11/21) in clade 9, ~46.4% (51/110) in clade 5, ~40.4% (21/52) in clade 17, 4.7% in clade 14 and 3.0% (4/133) in clade 8, whereas clade 11 is a singleton. Within clade 5 of the microviruses, the viruses are derived from various animals samples including capybara (*n* = 11), cow (*n* = 6), chimpanzee (*n* = 3), dog (*n* = 1), fish (*n* = 2), macaque (*n* = 3), mink (*n* = 4), moose (*n* = 2), ping (*n* = 19), rat (*n* = 1), tortoise (*n* = 51), yak (*n* = 6) (Supplementary Data 1). Whereas, within clade 17, the microviruses are derived from the bacterial species *Citromicrobium* sp. (*n* = 1), *Ruegeria pomeroyi* (*n* = 2) and various animal samples (ciona, *n* = 6; cow, *n* = 2; fish, *n* = 17; mouse, *n* = 1; nematode, *n* = 1; tortoise, *n* = 21, unknown animal, *n* = 1; Supplementary Data 1). 

Based on the identification of the 119 microviruses and the large number of unclassified ones, it is evident that these viruses, like CRESS DNA viruses, are highly diverse, found in various sample types and appear to have variants in their gene order within the genomes. It is also evident that the taxonomy of these viruses needs to be more thoroughly assessed.

## 4. Conclusions

In this study, using 40 fecal samples from Sonoran Desert tortoises collected in Arizona (USA), we identified 156 novel viruses, including 27 genomoviruses, 10 unclassified CRESS viruses, and 119 microviruses. The genomoviruses and microviruses likely infect organisms in the diet or gut of the tortoise, whereas diverse unclassified viruses may infect the tortoise themselves or organisms associated with them. Without a doubt, further studies would need to be carried out to determine the infectivity of these unclassified viruses in tortoise tissue or blood samples. Nonetheless, here, we highlight the high diversity of ssDNA viruses in the Sonoran Desert tortoise fecal matter.

While some pathogens, such as the bacterial *Mycoplasma* infection causal to upper respiratory tract disease in tortoises, have been well studied, other than this study, there have been no others that have attempted to evaluate their viral diversity. Further studies are required to elucidate whether these novel viruses are associated with the tortoises themselves or associated with diet and the desert environment.

## Figures and Tables

**Table 1:** Summary of the rolling circle replication (RCR) endonuclease motifs (Motif I, II, II) and superfamily 3 (SF3) helicase motifs (Walker A, Walker B, and Motif C) encoded genomoviruses, unclassified CRESS DNA viruses and Rep-encoding circular molecules identified in this study.

		Rolling Circle Replication (RCR) Endonuclease			Superfamily 3 (SF3) Helicase Motifs		
Genus/Group	Accession #	Motif I	Motif II	Motif III	Walker A	Walker B	Motif C
Gemykolovirus	MK570209	MLTYSD	PHFHC	RRWDYVGK	GATRLGKTVWAR	VFDDI	WLCN
	MK570213	FLTYSN	PHFHC	RRWDYVGK	GETRLGKTVWAR	IFDDI	WICN
Gemykibivirus	MK570202	LLTYPQ	VHLHA	KGAAYAIK	GGTRLGKTLWAR	VFDDM	YISN
	MK570214	LLTYPQ	VHLHA	KGAAYAIK	GGTRLGKTLWAR	IFDDM	YISN
	MK570215	LLTYPQ	VHLHA	KGAAYAIK	GGTRLGKTLWAR	IFDDM	YISN
	MK570205	LLTYPQ	IHLHA	KGYAYAIK	GPTRLGKTLWAR	VFDDM	YISN
	MK570211	LLTYPQ	VHLHA	KGYAYAIK	GPTRLGKTLWAR	VFDDM	YISN
	MK570216	LLTYPQ	VHLHA	KGYAYAVK	GPTRLGKTLWAR	VFDDM	YISN
	MK570207	LLTYPQ	IHLHA	KGYAYATK	GPTRLGKTLWAR	VFDDM	YISN
	MK570208	LLTYPQ	VHLHA	KGYAYAIK	GPTRLGKTLWAR	VFDDM	YISN
	MK570201	LLTYPQ	IHLHA	KGYAYAIK	GPTRLGKTLWAR	IFDDM	YISN
	MK570203	LLTYPQ	VHLHA	KGYAYAIK	GPTRLGKTLWAR	IFDDM	YISN
	MF373640	LLTYPQ	VHLHA	KGYAYAIK	GPTRLGKTLWAR	VFDDM	YISN
	MK570206	LLTYPQ	VHLHA	KGYAYAIK	GPTRLGKTLWAR	VFDDM	YISN
	MF373641	LLTYPQ	VHLHA	KGYAYAIK	GPTRLGKTLWAR	VFDDM	YISN
	MF373638	LLTYPQ	VHLHA	KGYAYAIK	GPTRLGKTLWAR	VFDDM	YISN
	MF373639	LLTYPQ	VHLHA	KGYAYAVK	GPTRLGKTLWAR	VFDDM	YISN
Gemycircularvirus	MK570218	LITYAQ	VHLHA	KGYDYAIK	GDSQLGKTVWAR	IFDDM	WLCN
	MK570204	LLTYPQ	IHLHA	KGYDYAIK	GDSQLGKTVWAR	VFDDM	WLCN
	MK570212	LLTYPQ	FHLHA	KGYDYAIK	GDSQLGKTLWAR	VFDDM	WLCN
	MK570210	LLTYAQ	IHLHV	TMYDYAIK	GESRLGKTVWAR	VFDDM	WLAN
	MK570223	LFTYAQ	IHFHV	TAYDYACK	GPYGCGKTVWAR	IFDDW	WLCN
	MK570217	LITYSQ	VHLHC	KGWDYACK	GASQTGKTLWAR	VFDDI	WLSN
	MK570222	LITYSQ	IHLHC	KGWDYACK	GASQTGKTLWAR	VFDDI	WLSN
	MK570219	LITYSQ	IHLHC	KGYDYAIK	GASQTGKTLWAR	VFDDI	WLSN
	MK570220	LITYSQ	IHLHC	KGYDYAIK	GASQTGKTLWAR	VFDDI	WLSN
	MK570221	LLTYAQ	SHLHC	AGFDYACK	GEPLTGKTDWAR	IFDDI	WCAN
Unclassified CRESS DNA viruses	MK858252	FLTYPQ	DHLHA	DVYNYVIK	GPSKTGKTQWAR	VIDDM	ILCN
	MK858253	FLTYPQ	DHLHA	DVYNYVIK	GPSKTGKTQWAR	VIDDM	ILCN
	MK858254	FLTYPQ	DHLHA	DVYNYVTK	GPSKTGKTAWAR	VIDDM	ILCN
	MK858255	FLTYPQ	NHLHV	DVYAYITK	GASKTGKTQWAR	VLDDL	ILCN
	MK858256	FLTYPR	DHLHV	HVYRYVRK	GPSKTGKTEWAR	IFDDL	ILCN
	MK858257	FLTFAR	DHRHV	GARQYTQK	GPSKTGKTHWAR	VFDDL	ILCN
	MF373642	FLTYPQ	PHLHC	AVRRYCSK	GNTETGKTTLAK	ILDDM	ITSN
	MK858265	LVTWSQ	LHYHA	DALAYVKK	GPTGSGKTRCAI	IFDDM	-
	MK858262	CKKYRR	PHIQG	ECVTYCKK	GPSGVGKTREVE	VFDDF	RITN
	MK858258	-	-	-	GGSNTGKTTYLR	WIDEF	TLKN
Rep-encoding circular molecules	MK858259	CFTWNN	PHIQG	DNFKYCTK	GPAGTGKTTWGR	CIEDY	VTSN
	MK858260	CFTWNN	PHIQG	DNFKYCTK	GPAGTGKTTWGR	CIEDY	VTSN
	MK858263	LLTFNN	YHTHL	ENRAYVLK	GETGTGKTSSVM	LFDEF	LVSN
	MK858264	-	YHTHL	ENREYIRK	GSTGTGKTSYVM	LFDEF	IISN

**Table 2:** Pairwise identity comparisons (showing highest identity) of the genome, and the replication associated protein (Rep) and capsid protein (CP) amino acid sequences of the genomoviruses identified in this study against themselves and those available in GenBank.

		Genomoviruses from This Study						Other Genomoviruses					
	Query	Genome		Rep		CP		Genome		Rep		CP	
Genus	Accession	% ID	Accession #	% ID	Accession #	% ID	Accession #	% ID	Accession #	% ID	Accession #	% ID	Accession #
Gemykolovirus	MK570209	65.21	MK570213	69.97	MK570213	45.45	MK570221	70.08	MK939374	81.38	MK939374	49.16	KT862242
	MK570213	65.21	MK570209	69.97	MK570209	44.44	MK570223	66.96	MH545501	71.26	MK939374	47.42	MH545501
Gemykibivirus	MF373638	99.82	MF373641	99.69	MF373641	100.00	MF373641	99.22	MK249293	98.46	MK249293	99.67	MK249293
	MF373639	99.73	MF373641	99.69	MF373641	99.67	MF373640	99.31	MK249293	98.46	MK249293	100.00	MK249293
	MF373640	96.97	MF373638	97.23	MF373641	100.00	MF373641	96.87	MK249293	97.23	MK249293	99.67	MK249293
	MF373641	99.82	MF373638	99.69	MF373638	100.00	MF373638	99.22	MK249293	98.77	MK249293	99.67	MK249293
	MK570201	96.78	MF373639	98.77	MK570203	99.67	MF373639	96.84	MK249293	94.98	MK947374	99.67	MK249293
	MK570202	99.47	MK570214	99.08	MK570214	100.00	MK570214	90.70	MK249269	83.69	MK249269	98.38	MK249239
	MK570203	94.34	MK570201	98.77	MK570201	98.36	MK570208	95.39	MK249300	95.32	MK947374	99.34	MK249300
	MK570205	95.57	MK570216	95.08	MK570211	99.68	MK570215	97.41	MK249307	99.08	MK249307	98.70	MK249239
	MK570206	96.55	MF373639	99.38	MF373641	99.67	MK570211	91.00	MK947374	98.77	MK249293	98.36	MK249293
	MK570207	97.47	MK570208	97.23	MK570208	97.94	MK570208	92.44	MK249293	96.92	MK249293	97.25	MK249300
	MK570208	97.47	MK570207	97.23	MK570207	98.36	MK570203	92.94	MK249293	96.92	MK249293	98.36	MK249300
	MK570211	94.98	MK570206	95.08	MK570205	99.67	MK570206	92.59	MK249293	97.23	MK249269	98.03	MK249293
	MK570214	99.47	MK570202	99.69	MK570215	100.00	MK570202	90.62	MK249269	83.38	MK249269	98.38	MK249239
	MK570215	98.67	MK570214	99.69	MK570214	100.00	MK570216	90.35	MK249269	83.08	MK249269	99.03	MK249239
	MK570216	95.57	MK570205	94.77	MF373639	100.00	MK570215	94.14	MK249307	94.46	MK249236	99.03	MK249239
Gemycircularvirus	MK570204	81.28	MK570212	93.99	MK570212	61.76	MK570210	73.52	MK947372	82.53	MK947372	70.10	MG641202
	MK570210	67.50	MK570204	58.66	MK570212	61.76	MK570204	76.02	MK939384	92.42	MK939384	75.40	JQ412056
	MK570212	81.28	MK570204	93.99	MK570204	59.28	MK570218	74.28	MK947372	82.83	MK947372	69.81	MG571096
	MK570217	97.56	MK570222	94.80	MK570222	99.02	MK570222	68.13	KM821747	76.76	KJ547638	50.00	KM510192
	MK570218	80.78	MK570212	87.39	MK570204	59.28	MK570212	74.01	MK947372	80.72	MK947372	63.61	MG571100
	MK570219	97.12	MK570220	98.78	MK570220	99.03	MK570220	68.06	KM821747	74.31	KJ547638	53.87	MK939446
	MK570220	97.12	MK570219	98.78	MK570219	99.03	MK570219	67.97	MK939432	74.01	KJ547638	55.08	MK939446
	MK570221	66.65	MK570204	52.00	MK570210	50.33	MK570204	65.90	MK939384	66.03	MG641197	58.36	KT732806
	MK570222	97.56	MK570217	96.94	MK570219	99.02	MK570217	67.91	KM821747	74.62	KM821747	50.43	KM510192
	MK570223	61.64	MK570210	50.76	MK570210	45.54	MK570204	82.49	MG571087	94.22	MG571087	70.39	KF413620

**Table 3:** Summary of recombination events identified in the genomes of gemycircularviruses and gemykibiviruses from this study. Major and minor parents indicate sequences (GenBank accession # provided) related to parental sequences that respectively donated the larger and smaller regions of the recombinant genome. For each event the recombination detection method with the most significant associated *p*-value is indicated in bold. Recombination detection methods: RDP (R), GENCONV (G), BOOTSCAN (B), MAXCHI (M), CHIMERA (C), SISCAN (S) and 3SEQ (T). Sites where the actual breakpoint is undetermined are marked with *.

Event	Begin	End	Recombinant Sequence(s)	Minor Parental Sequence(s)	Major Parental Sequence(s)	*p*-Value	Method
I	190	1017	MK570210	Unknown	MH939384	2.71 × 10^−25^	MCS
II	249 *	1050	MK570204, MK570212, MK570218	Unknown	MK570204	6.55 × 10^−16^	GBMCST
III	243 *	1009	MK570218	Unknown	MK570204	5.10 × 10^−14^	GBMCT
IV	1539 *	1591	MK570222, MK570217	MF173067	MK570220, MK570219	7.12 × 10^−8^	RGBT
V	23	1144	MK570202, MK570214, MK570215	MK249302, MK249239, MK249243, MK249246, MK249252, MK249256, MK249268, MK249269, MK249275, MK249287, MK249288, MK249307, MK570205, MK570216	Unknown	1.67 × 10^−52^	RGBMCST
VI	1275	2192	MK570211	MK249239, MK249243, MK249246, MK249252, MK249256, MK249268, MK249269, MK249275, MK249287, MK249288, MK249302, MK249307	MF373638, MF373639, MF373640, MF373641, MK249293, MK570206	3.64 × 10^−86^	GBMCST
VII	333	1113	MF373638, MF373639, MF373640, MF373641, MK570201, MK570203, MK570206, MK570207, MK570208, MK570211	MK483084	MH973737, MH973738, MH973739, MH973740, MK249236, MK249241, MK249251, MK249255, MK249271, MK249276, MK249279, MK249286, MK249301	7.69 × 10^−30^	GBMCST
VIII	1643	1958	MK570216	MH973737, MH973738, MH973739, MH973740, MK249236, MK249241, MK249251, MK249255, MK249271, MK249276, MK249279, MK249286, MK249293, MK249301, MK570206	MK249239, MK249243, MK249246, MK249252, MK249256, MK249268, MK249269, MK249275, MK249287, MK249288, MK249302, MK249307, MK570205	5.32 × 10^−25^	RGBMCST
IX	1993	2211	MK570205, MK570216	MF373638, MF373639, MF373640, MF373641, MH973737, MH973738, MH973739, MH973740, MK249236, MK249241, MK249251, MK249255, MK249271, MK249276, MK249279, MK249286, MK249293, MK249296, MK249301, MK570206, MK947374	MK249239, MK249243, MK249246, MK249252, MK249256, MK249268, MK249269, MK249275, MK249287, MK249288, MK249302	7.83 × 10^−22^	GBMCST

**Table 4:** Pairwise identities (showing highest identity) of the replication associated protein (Rep) and capsid protein (CP) amino acid sequences of the unclassified CRESS DNA viruses and circular molecules identified in this study against themselves and against those available in GenBank.

		Unclassified CRESS DNA Viruses from This Study				Other Unclassified CRESS DNA Viruses			
	Query	Rep		CP		Rep		CP	
Virus Group/Molecules	Accession #	Accession #	%ID	Accession #	%ID	Accession #	%ID	Accession #	%ID
Unclassified CRESS DNA virus	MF373642	MK858254	29.76	-	-	KY487833	42.00	-	-
	MK858252	MK858253	100.00	MK858253	99.00	MK570182	78.42	MK858254	38.68
	MK858253	MK858252	100.00	MK858253	99.00	MK570182	78.42	MK858254	38.68
	MK858254	MK858252	81.16	MK858255	39.16	MK570182	75.99	MK570182	39.25
	MK858255	MK858252	79.03	MK858254	39.25	MK570182	83.57	MK570165	53.64
	MK858256	MK858255	65.75	MK858257	33.47	MK570182	64.44	MK570168	33.46
	MK858257	MK858256	56.23	MK858254	25.21	MK570165	54.08	MK570168	42.45
	MK858258	MK858260	35.57	MK858261	52.65	MK570170	45.86	-	-
	MK858262	MK858265	30.80			MH616676	46.77	KY302869	28.12
	MK858265	MK858262	30.80	-	-	KM821751	30.80	-	-
Circular molecule	MK858259	MK858260	99.70	-	-	MF118167	49.34	-	-
	MK858260	MK858259	99.70	-	-	MF118167	49.34	-	-
	MK858261	-	-	MK858258	52.65	-	-	-	-
	MK858263	MK858264	72.53	-	-	MH616676	47.17	-	-
	MK858264	MK858263	72.53	-	-	MH616676	47.17	-	-

## Supplementary Materials

The following are available online at https://www.mdpi.com/1999-4915/12/2/143/s1, Supplementary Figure 1: Approximately Maximum-Likelihood cladogram of the MCP sequences (*n* = 2797). Branches are color coded based on sub-families (*Bullavirinae* and *Gokushovirinae*) and Alphaviriniae-, Parabacterioides- and Pichovirinae-clades. In addition to these, 22 unique clades are marked with number. Branches in grey represent an additional nine singletons and 13 clades. Branches in red denote sequences identified in this study. The outer circle represents taxa with some level of classification assigned prior to this study. Branch support with >0.8 aLRT is shown. Taxa names and assignment are provided in Supplementary Data 1. Supplementary Figure 2: Approximately Maximum-Likelihood phylogram of the MCP sequences (*n* = 2797). Branches are color coded based on sub-families (*Bullavirinae* and *Gokushovirinae*) and Alphaviriniae-, Parabacterioides- and Pichovirinae-clades. Branches in grey represent an additional nine singletons and 13 clades. Branches in red denote sequences identified in this study. The outer circle represents taxa with some level of classification assigned prior to this study. Branch support with >0.8 aLRT is shown. Taxa names and assignment are provided in Supplementary Data 1. Supplementary Table 1: Details of abutting primers used to recover the full genomes of genomoviruses and unclassified CRESS DNA viruses for this study. Supplementary Data 1: Summary of microviruses whose MCPs were used to generate the phylogeny presented in Figure 5 and Supplementary Figures S1 and S2. Detailed of accession number, sub-family/putative sub-families (based on phylogeny presented in this study), genome length, country of identification, and isolation source are provided.
