# Supplementary material for: Virus Discovery in Desert Tortoise Fecal Samples: Novel Circular Single-Stranded DNA Viruses
Source: Viruses. 2020 Jan 26;12(2):143. doi: 10.3390/v12020143 (PMC7077246; doi:10.3390/v12020143)
Supplement: Supplementary file 1 [file viruses-12-00143-s001.zip › Supplementary table 1.docx]

| **Genus / group** | **Accession #** | **Forward** | **Reverse** |
| --- | --- | --- | --- |
| **Gemykolovirus** | MK570209 | ATTGATATCGTCGAATACTGCGTACTTGCAAT | GGAGGATTTGGATACTTTCCTTCTTACAAGCA |
|  | MK570213 | CCACCGCTCGGGATAAAACTAC | GACACCATATGAGCTGATAACCCTTATC |
| **Gemykibivirus** | MK570202 | CAGGGAGGGATTAAGTTCTTTCATTCATAC | CATATCGTCGAAAACTGCGTAGTC |
|  | MK570214 | GGAGGGATTAAGTTCTTTCATGCATAC | CTGCATATCGTCGAAAATGGCATAG |
|  | MK570215 | GTCCAGTAGCATCAATACTTAATACATCCG | GATTCTACTGGCATGAGAAGTGATTCTG |
|  | MK570205 | CAGGGAGGGATTAAGTTCTTTCATTCATAC | CATATCGTCGAAAACTGCGTAGTC |
|  | MK570211 | CAGGGAGGGATTAAGTTCTTTCATTCATAC | CATATCGTCGAAAACTGCGTAGTC |
|  | MK570216 | GTCCAGTAGCATCAATACTTAATACATCCG | GATTCTACTGGCATGAGAAGTGATTCTG |
|  | MK570207 | GTTAATGCCATGTTTCCGGAAAATGTC | GATGATCAGTCTACTCTAACCTTCACTC |
|  | MK570208 | GTTAATGCCATGTTTCCGGAAAATGTC | GATGATCAGTCTACTCTAACCTTCACTC |
|  | MK570201 | GTTAATGCCATGTTTCCGGAAAATGTC | GATGATCAGTCTACTCTAACCTTCACTC |
|  | MK570203 | GTTAATGCCATGTTTCCGGAAAATGTC | GATGATCAGTCTACTCTAACCTTCACTC |
|  | MF373640 | CTTCGGAAGTAGGCGGAAGTGTCG | GACCAGTAACGGCAGGGTCCGTCTC |
|  | MK570206 | GTTAATGCCATGTTTCCGGAAAATGTC | GATGATCAGTCTACTCTAACCTTCACTC |
|  | MF373641 | CTTCGGAAGTAGGCGGAAGTGTCG | GACCAGTAACGGCAGGGTCCGTCTC |
|  | MF373638 | CTTCGGAAGTAGGCGGAAGTGTCG | GACCAGTAACGGCAGGGTCCGTCTC |
|  | MF373639 | CTTCGGAAGTAGGCGGAAGTGTCG | GACCAGTAACGGCAGGGTCCGTCTC |
| **Gemycircularvirus** | MK570218 | GCAACGCCTATAAATACCTGTCACTC | CTGTCCCTGTCCCTTTTGCTATAATATTAG |
|  | MK570204 | GATCCAGATCTTGCCCTAAACATATCA | TTCGACGACTGATCAACTTCAAGTTAG |
|  | MK570212 | GCAACGCCTATAAATACCTGTCACTC | CTGTCCCTGTCCCTTTTGCTATAATATTAG |
|  | MK570210 | CTTTCTTAGGCCAAAGTCTCTTGTCCTGTAC | CTTCAATTAGCATGAGTCACAGCAAGGAGTG |
|  | MK570223 | AGAACCATGAACAATATCAACACGAAGTCATCATC | GACCATTCCATTTGAAGTCTCGGTGTAGTAGTTC |
|  | MK570217 | GGTTGGGATTATGCATGCAAAGATGGAGATATCG | CTTTTCTGGTGTTCCTTTAGACTGCTCAATATTGG |
|  | MK570222 | GGTTGGGATTATGCATGCAAAGATGGAGATATCG | CTTGTCTGGTGTTCCTTTAGACTGGACAATATTGG |
|  | MK570219 | GGCTACGATTATGCAATCAAAGATGGAGATATCG | CTTGTCTGGTGTTCCTTTAGACTGGACAATATTGG |
|  | MK570220 | GGCTACGATTATGCAATCAAAGATGGAGATATCG | CTTGTCTGGTGTTCCTTTAGACTGGACAATATTGG |
|  | MK570221 | GGAGGTATCAAATTCTTTCATGGGTTC | GCGGATGTCGTCAAAAATAGCATATTC |
| **Unclassified CRESS DNA viruses** | MK858252 | GTTACGTGATTTGTATAACCCAGGTG | GGAATAATTGAATATGCCCCTGAGAC |
|  | MK858253 | GTTACGTGATTTGTATAACCCAGGTG | GGAATAATTGAATATGCCCCTGAGAC |
|  | MK858254 | CTCTAAAGAACATATGCCTACCTAATGACC | AGAATTTCAACCTAGACGATTGGGATC |
|  | MK858255 | ACCCATACTACCTAAAAGGACCTTC | GACATGGTATTATACGATAGATATAGGCCC |
|  | MK858256 | ACTGGCGGTATAGTGAAATATGGAAG | CTACTCCGGGGTTACAAAGTATTATACAAC |
|  | MK858257 | TATCAGTCAATGGAGTAACGGGTACATAG | ACTATCCAGTAGTAATTCCATCCCTTCAG |
|  | MF373642 | AGTGCGACCTCCCCAGGGAAGAG | GAGGGTACGTCAGGAAGAGTGTCTTC |
|  | MK858265 | TATTCTGACTGGTAGGTCGTATCACTAAC | CTAGGAGGAATATCTATAGCTTCAAGGAC |
|  | MK858262 | GTCCTCGTACATATAGACGACGTATTC | GATATCGACGTCTATATGTACGCTTACG |
|  | MK858258 | CTGTAGTTACCTGTGTAAAGACTTGATGG | GAGATATAGAAGAGCATTTAGGCGATATGG |
| **Rep encoding circular molecules** | MK858259 | CGGTCAGTCTTCAAGTATTTGCTCAAATATGAACC | TTTTGAGATCACTGGAGAATATAAAGGTGGTTCGG |
|  | MK858260 | ACCGTTTTGAGATCACTGGAGAATATAAAGGT | CACCCCAGTCCTTCAAGTATTTGCTCAAATAT |
|  | MK858263 | TATGCCTTTTACCACGACTGTCAACATAGTCATAG | CGGGCATAAACTATTCTGATACCTTTGAGGAAAG |
|  | MK858264 | ACCACCTGCATGAAAGGAGTTTATTATGACAG | TTCAGCCTGACGTTATTGATTGACAACAGTAT |
| **Non-Rep encoding circular molecule** | MK858261 | CTCCCGGTACTACAGATATATTTTCAGTG | CGACACCTTGATATACTTCTTGTGTTG |
