# Supplementary figures and images for: Virus Discovery in Desert Tortoise Fecal Samples: Novel Circular Single-Stranded DNA Viruses
Source: Viruses. 2020 Jan 26;12(2):143. doi: 10.3390/v12020143 (PMC7077246; doi:10.3390/v12020143)

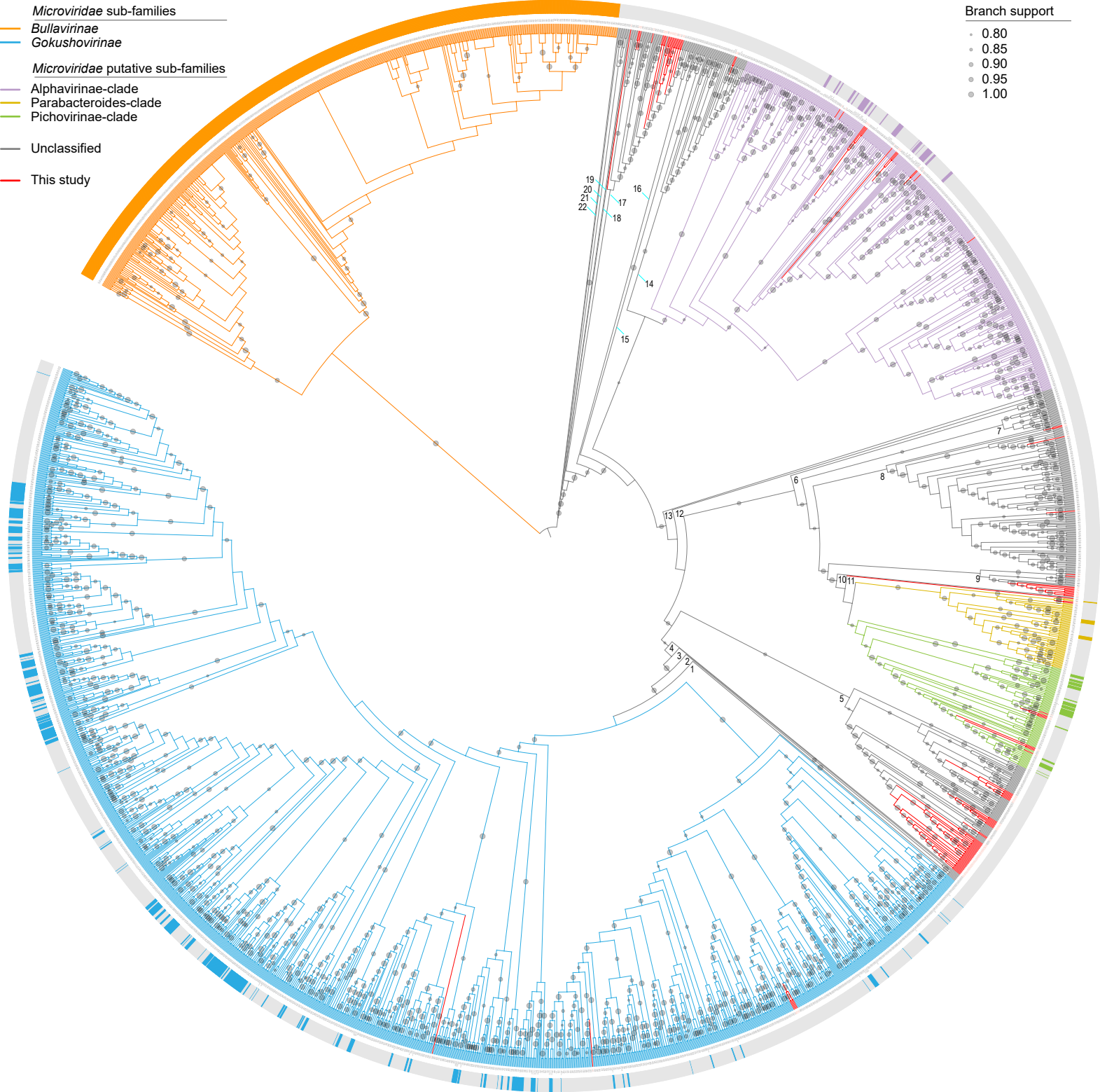

Supplement: Supplementary file 1 [file viruses-12-00143-s001.zip › Supplementary Figure 1.pdf]

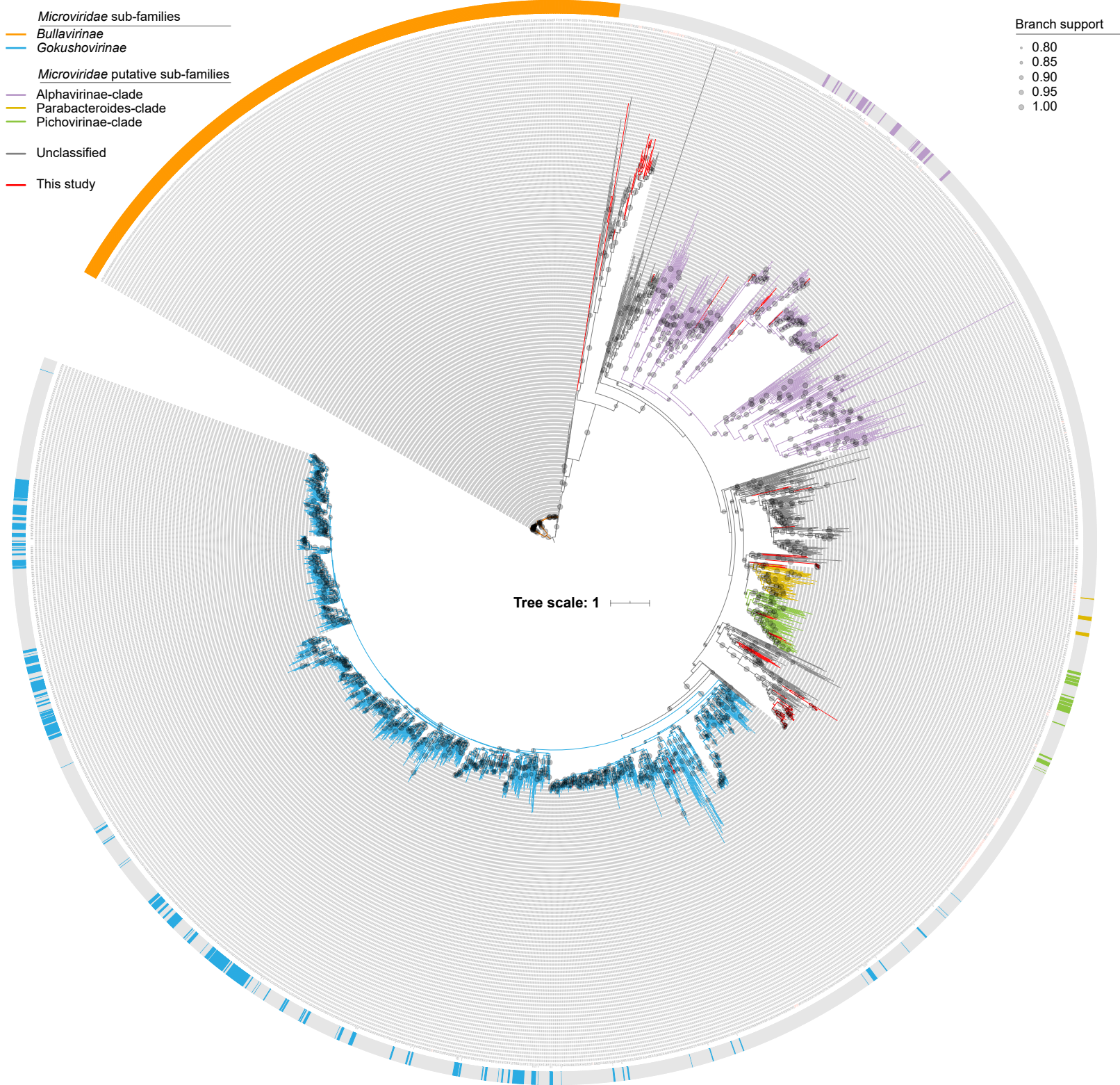

Supplement: Supplementary file 1 [file viruses-12-00143-s001.zip › Supplementary Figure 2.pdf]
